# Mental ill-health and substance use among sexuality diverse adolescents: The critical role of school climate and teacher self-efficacy

**DOI:** 10.1177/00048674231202427

**Published:** 2023-09-29

**Authors:** Sasha Bailey, Nicola C Newton, Yael Perry, Ashleigh Lin, Lucinda Grummitt, Emma L Barrett

**Affiliations:** 1 The Matilda Centre for Research in Mental Health and Substance Use, Faculty of Medicine and Health, The University of Sydney, Sydney, NSW, Australia; 2 Telethon Kids Institute, University of Western Australia, Perth, WA, Australia; 3 The University of Western Australia, Perth, WA, Australia

**Keywords:** Sexuality diversity, adolescence, mental health, substance use, schools

## Abstract

**Introduction:**

Mental ill-health, substance use and their co-occurrence among sexuality diverse young people during earlier adolescence is relatively understudied. The preventive utility of positive school climate for sexuality diverse adolescents’ mental health is also unclear, as well as the role of teachers in conferring this benefit.

**Method:**

Using Wave 8 ‘B Cohort’ data from the Longitudinal Study of Australian children (*N* = 3127, *M*_age_ = 14.3), prevalence ratios and odds ratios were used to assess prevalence and disparities in mental ill-health and substance use, and multinomial logistic regression for co-occurring outcomes, among sexuality diverse adolescents relative to heterosexual peers. Logistic regression was used to assess associations between school climate and teacher self-efficacy with sexuality diverse adolescents’ mental health.

**Results:**

Mental ill-health prevalence ranged from 22% (suicidal thoughts/behaviour) to 46% (probable depressive disorders) and substance use between 66% (cigarette use) and 97% (alcohol use). Sexuality diverse participants were significantly more likely to report self-harm and high levels of emotional symptoms in co-occurrence with cigarette, alcohol and/or cannabis use. For each 1-point increase in school climate scores as measured by the Psychological Sense of School Membership scale, there was 10% reduction in sexuality diverse adolescents reporting high levels of emotional symptoms, probable depressive disorder, self-harm thoughts/behaviour and suicidal thoughts/behaviour. For each 1-point increase in lower perceived (worse) teacher self-efficacy scores as measured by four bespoke teacher self-efficacy items, odds of sexuality diverse adolescent-reported suicidal thoughts/behaviour increased by 80%.

**Discussion:**

Mental ill-health, substance use and especially their co-occurrence, are highly prevalent and pose significant and inequitable health and well-being risks. Schools represent a potential site for focusing future prevention efforts and educating and training teachers on sexuality diversity is a promising pathway towards optimising these.

## Introduction

Sexuality diverse young people, that is, young people who do not identify as heterosexual, who are not exclusively attracted romantically and/or sexually to those of ‘the other’ gender, do not have feelings of romantic and/or sexual attraction or feel an affinity to a sexuality diverse community, bear significantly disproportionate burden of mental ill-health, such as depression and anxiety disorders, and substance use, such as tobacco smoking, alcohol consumption and illicit drug use compared with their heterosexual counterparts (Hill et al., 2021a, 2021b; Robinson et al., 2014). This is a particularly urgent public health issue given mental ill-health and substance use often co-occur and share common, interacting risk factors, which typically develop and onset during adolescence, leading to increased risk of chronic, debilitating mental and substance use disorders throughout the lifespan (Solmi et al., 2022; Stockings et al., 2016). This is in addition to those risk factors present from birth, such as family genetics (Stockings et al., 2016), and early childhood, such as adverse childhood experiences (Kessler et al., 2010).

Despite evidence suggesting mental health and substance use disparities are present among sexuality diverse young people relative to heterosexual peers, previous research has largely investigated disparities across sexuality diverse young people as an umbrella age group, conflating adolescence and young adulthood (Hill et al., 2021b). Furthermore, available epidemiological evidence regarding mental ill-health and substance use prevalence among sexuality diverse adolescents often utilises convenience sampling methods, excludes heterosexual comparators or lacks sufficient sample size and/or statistical power (Meyer et al., 2021; Turban et al., 2023). Many large-scale paradigm-shifting studies have been undertaken to better understand mental ill-health and substance use among sexuality diverse adolescents in Australia (Elizabeth et al., 2014; Hill et al., 2021b; Robinson et al., 2014). These large, unprecedent sample sizes have necessarily employed community-engaged convenience sampling method to reach the largest number of sexuality diverse young people (Hill et al., 2021b; Elizabeth et al., 2014; Robinson et al., 2014). To our knowledge, there are no population-level, nationally representative estimates of mental ill-health and substance use among sexuality diverse adolescents in Australia. Furthermore, available literature in Australia and worldwide often considers mental ill-health and substance use among sexuality diverse adolescents in a siloed manner (Hill et al., 2021b). The common co-occurrence and overlapping aetiologies of mental ill-health and substance use thus highlights an urgent research gap: quantifying the prevalence of co-occurring mental ill-health and substance use among sexuality diverse adolescents.

School climate, that is, the effects of a school’s academic atmosphere, community of interpersonal relationships, physical and emotional safety and institutional structures, is a powerful determinant of mental ill-health and substance use among sexuality diverse young people (Fantus and Newman, 2021; Wang and Degol, 2016). Negative experiences within the school climate due to heterosexist bullying, discrimination and harassment from peers and staff are more often reported by sexuality diverse young people, compared with their heterosexual peers (Day et al., 2018, 2020). Emerging evidence highlights that positive school climate can prevent and reduce mental ill-health among sexuality diverse young people (Colvin et al., 2019; Leung et al., 2022). This research, however, does not indicate whether this benefit extends to sexuality diverse adolescents during early adolescence as opposed to sexuality diverse young people during early adulthood. Moreover, a substantial portion of available evidence operationalises school climate as sexuality diverse school activities, such as gender sexuality/gay–straight alliances or anti-bullying programmes (Colvin et al., 2019; Parodi et al., 2022). These measures fail to capture peer, teacher and environmental influences within school climate, which likely play an important role in sexuality diverse mental ill-health.

One school factor garnering interest in leveraging the preventive utility of school climate is teacher self-efficacy, that is, the self-perceived capacity to produce desired educational outcomes and experiences for students (Lauermann and König, 2016). An important facet of this is the management of behavioural problems (Lauermann and König, 2016). This is particularly relevant to addressing the burden of mental ill-health and substance use among sexuality diverse young people given this population experiences alarming rates of bullying and discrimination (Hill et al., 2021b), often without positive adult intervention (Ullman, 2021). There is a significant lacuna of evidence regarding the associations between teacher self-efficacy and mental ill-health among sexuality diverse adolescents.

Using nationally representative, population-based data from The Longitudinal Study of Australian Children (LSAC), this study aims to address these research gaps through four aims: (1) to assess prevalence and disparities of mental ill-health and substance use among sexuality diverse adolescents, compared to their heterosexual peers; (2) to assess disparities of co-occurring mental ill-health and substance use among sexuality diverse adolescents, compared with their heterosexual peers; (3) to assess associations between sexuality diverse adolescents’ mental ill-health, substance use and co-occurring outcomes, with adolescent-reported school climate and (4) teacher-reported teacher self-efficacy.

## Methods

### Study design and participants

The LSAC is a cross-sequential study comprising two 12-month age cohorts (B cohort, infants aged 0–1 years, and K cohort, children aged 4–5 years old). This study used data from Wave 8 of the B cohort, who were born between March 2003 and February 2004 (aged 3–15 months; Wave 1) and were followed up in 2018 when they were aged 14–16 years. The Wave 8 response rate was approximately 77.3% with a 61.1% retention rate from Wave 1 of the study (Department of Social Services, 2022). Verbal participant consent was captured. Full detail regarding the use of non-probability-based selection of participations via geographically representative postcode sampling with homogeneous probability of selection is published elsewhere (Soloff et al., 2005). LSAC methodology and survey content has received ethical review and approval by the Australian Institute of Family Studies Ethics Committee, a National Health and Medical research Council registered Human Research Ethics Committee. Participants consent to each individual LSAC wave of data collection and may withdraw from the study at any time or choose not to take part in some aspects of the study.

### Measures

#### Sexuality diversity

A dichotomous ‘Sexuality Diversity’ variable was computed for males and females who responded that they were attracted only to boys or girls, respectively, attracted to both girls and boys, were unsure who they were attracted to, or did not feel any attraction to others. Participants who met this criteria were coded 1 (Yes) and those who did not, 0 (No).

#### Mental ill-health

##### High levels of emotional symptoms

Participants responded to the self-report Strengths and Difficulties Questionnaire Emotional Symptoms sub-scale (Goodman, 2001), consisting of five items, which assessed frequency of internalising symptoms within the past 6 months, for example, often unhappy, many worries. Total scores were summed (ranging 0–10) with scores ⩽ 6 indicating normal or borderline levels of emotional symptoms, and scores ⩾ 7 indicating clinically significant levels of emotional symptoms (Goodman et al., 2000).

##### Probable depressive disorder

The 13-item Short Moods and Feelings Questionnaire (Angold et al., 1995; SMFQ) was used to assess past month depressive symptomatology among participants, comprising 13 statements such as ‘I felt nobody loved me’. Participants responses were summed (ranging 0–26) and scores of 8 or higher were defined as indicative of a probable depressive disorder (Daraganova, 2017). The SMFQ has been previously validated as a sound self-report depression checklist of core symptoms for children and adolescents aged 8–16 (Turner et al., 2014).

##### Self-harm ideation and attempts

Participants indicated ‘yes’ or ‘no’ to whether in the past 12 months they had ‘thought about hurting yourself on purpose in any way?’ or ‘hurt yourself on purpose in any way?’. Participants that responded ‘yes’ to either question were coded as reporting self-harm thoughts/behaviours.

##### Suicidal ideation, planning and attempts

Participants were asked (yes/no) whether during the past 12 months they had ‘ever seriously consider attempting suicide?’ or ‘made a plan about how you would attempt suicide?’ Participants were also asked ‘During the past 12 months, how many times did you actually attempt suicide? (0 = 0 times, 1 = 1 time, 2 = 2 or 3 times, 3 = 4 or 5 times, 4 = 6 or more times). Suicide attempts were categorised and dichotomised where ⩾ 1 indicated a past 12 month suicide attempt, and 0 indicated no past 12 month suicide attempt. Participants who responded ‘yes’ to suicidal ideation or planning items, or reporting ⩾ 1 suicide attempts, were coded as indicating suicidal thoughts/behaviours.

#### Substance use

##### Recent cigarette, alcohol and cannabis use

Participants were asked (yes/no) whether during the past 12 months they had used cigarettes, alcohol or cannabis.

#### Co-occurring mental ill-health and substance use

In accordance with a previous study of co-occurring depressive symptoms and probable substance use disorders among sexuality diverse young people (Felner et al., 2021), the four mental ill-health variables (probable anxiety/depressive disorder, probable depressive disorder, self-harm thoughts/behaviours and suicidal thoughts/behaviour) were combined with three substance use variables (recent cigarette use, recent alcohol use, recent cannabis use) to create 12 co-occurring mental ill-health and substance use variables. Each co-occurring variable comprised four levels: neither mental ill-health or substance use outcome present; only mental ill-health outcome present, only substance use outcome present, both mental ill-health and substance use outcome present.

#### School climate

Twelve items were used from the 18-item validated Psychological Sense of School Membership (PSSM) scale, designed and previously validated as a sound measure of adolescents’ perceived belonging or psychological membership in the school environment (Goodenow, 1993). Responses to individual items range from 1 (Not at all true) to 5 (Complete true) across 12 statements, such as ‘I can be myself at school’ and ‘I wish I was at a different school’ with total scores ranging 12–60 (negative items were reverse scored). Past research has found that 12-item PSSM has good reliability in the LSAC data set (Mooney et al., 2023; Spence et al., 2022).

#### Teacher self-efficacy

Administered to the teachers of students participating in the LSAC study, four researcher developed items (e.g. ‘I am competent with behavioural problems’) assessed teacher self-efficacy, that is, their self-perceived abilities to promote academic achievement and success and prevent behavioural and learning problems. Previous use of this teacher self-efficacy scale has found these items are internally valid and sound (alpha = 0.79; Dempsey, 2009). Higher scores indicated lower levels of teacher self-efficacy or, in other words, higher teacher self-efficacy scores indicated worse teacher self-efficacy perceptions.

### Statistical analysis

For descriptive statistics, categorical variables were summarised using counts and proportions and continuous variables, with means and standard deviations.

Prevalence ratios and odds ratios derived from logistic regression models were calculated to assess prevalence and disparities in mental ill-health and substance use among sexuality diverse adolescents compared with non-sexuality diverse adolescents. Multinomial logistic regression models were used to test disparities in co-occurring outcomes among sexuality diverse adolescents relative to non-sexuality diverse adolescents. Logistic regression models were used to assess associations between school climate and teacher-reported teacher self-efficacy, with sexuality diverse adolescents’ mental health outcomes. Mental ill-health variables and school climate items had < 10% missing data; however, substance use items had > 10% missing data. This was system missing error due to items not being asked to participants for routine reasons, for example, a question was skipped due to the answer to a preceding question. Teacher self-efficacy items generated > 10% missing data; however, due to lack of teacher-report demographic variables, it was difficult to assess underlying reasons for this missingness. To account for missing data, complete case analysis was conducted for all models.

All analyses utilised Wave 8 sample weights to correct for between-wave participant attribution and realign sample characteristics with the original representative probability sample design study. All analyses were conducted in R Studio Version 3.2.3.

## Results

Among the 3127 participants in this study (*M*_age_ = 14.3, SD_age_ = 0.5), 2503 (84.0%) identified as heterosexual/straight, and 475 (15.95%) identified as sexuality diverse. More detail regarding the demographic characteristics of sexuality diverse adolescents and their non-sexuality diverse comparators is available in Table 1.

**Table 1.** Demographic characteristics of young people by sexuality diversity.

	Non-sexuality diverse	Sexuality diverse
All (*N*)	2503	475
Age, *M* (SD)	14.3 (0.47)	14.3 (0.46)
Binary sex recorded at birth		
Female, *N* (%)	1142 (45.63)	307 (64.63)
Male, *N* (%)	1361 (54.37)	168 (35.37)
Indigenous status		
Aboriginal and/or Torres Strait Islander, *N* (%)	60 (2.40)	12 (2.53)
Australian state of residence, *N*		
NSW	754	147
VIC	588	102
QLD	575	110
SA	165	22
WA	261	60
TAS	76	*
NT	26	*
ACT	58	21
Region of residence		
Non-metropolitan	1023 (40.97)	166 (35.02)
Metropolitan	1474 (59.03)	308 (64.98)
Index of Relative Socio-economic Disadvantage Scores – *M* (SD)	1018 (64.1)	1017 (72.5)
Teacher data		
Teacher data available at Wave 6 data collection	2421 (96.7)	336 (70.1)

SD: standard deviation.

* It is a requirement by Australian Institute of Family Studies, the custodian of the Longitudinal Study of Australian Children, to censor cell values with counts less than 10.

### Mental ill-health prevalence and disparities

Table 2 reports prevalence of mental ill-health, substance use and co-occurring outcomes among sexuality diverse adolescents in the sample. Among sexuality diverse adolescents in this sample, 24% reported high levels of emotional symptoms, 46% reported probable depressive disorders, 32% reported past 12-month self-harm thoughts or behaviours, and 22% reported past 12-month suicidal thoughts or behaviours. Sexuality diverse adolescents had statistically significant higher odds of high levels of emotional symptoms, depressive disorders, self-harm thoughts/behaviours and suicidal thoughts/behaviours compared with their heterosexual peers (odds ratios (ORs) ranged 2.3–3.0).

**Table 2.** Prevalence of mental ill-health and substance use among adolescents by sexuality diversity.

	Sexuality diverse vs non-sexuality diverse			Sexuality diverse females vs non-sexuality diverse females			Sexuality diverse males vs non-sexuality diverse males		
	Non-sexuality diverse	Sexuality diverse		Non-sexuality diverse females	Sexuality diverse females		Non-sexuality diverse males	Sexuality diverse males	
	*N* %	*N* %	OR 95% CI *p*	*N* %	*N* %	OR 95% CI *p*	*N* %	*N* %	OR 95% CI *p*
High levels of emotional symptoms	241 9.6	115 24.4	3.0 2.26–4.04 *<* *0.001*	194 16.9	92 30.9	2.2 1.6–3.1 *<* *0.001*	47 3.5	23 13.2	4.2 2.21–8.10 *<* *0.001*
Probable depressive disorder	673 27.1	218 46.2	2.3 1.85–2.89 *<* *0.001*	387 33.8	165 55.5	2.4 1.8–3.3 *<* *0.001*	286 21.3	53 30.4	1.6 1.08–2.40 *0.018*
Recent self-harm thoughts/behaviours	370 14.9	149 31.8	2.7 2.07–3.42 *<* *0.001*	250 22.0	124 42.1	2.6 1.9–3.5 *<* *0.001*	119 8.9	25 14.4	1.7 1.04–2.84 *0.034*
Recent suicidal thoughts/behaviours	272 11.0	103 21.9	2.3 1.69–3.06 *<* *0.001*	161 14.1	80 27.0	2.2 1.6–3.2 *<* *0.001*	111 8.3	23 13.3	1.7 0.99–2.87 *0.055*
Recent cigarette use	168 68.0	29 66.3	0.9 0.38–2.27 *0.864*	85 73.7	25 73.3	1.0 0.3–3.5 *0.972*	84 63.0	*	*
Recent alcohol use	372 93.7	61 96.5	1.8 0.50–6.74 *0.36*	164 94.8	50 97.2	2.0 0.4–10 *0.42*	207 92.8	11 93.0	1.0 0.11–9.17 *0.983*
Recent cannabis use	129 79.7	23 91.1	2.6 0.71–9.60 *0.15*	48 76.2	19 96.8	9.6 1.1–85.4 *0.04*	81 81.9	*	*

OR: odds ratio; CI: confidence interval.

Italicised figures denote corresponding *p*-values.

* It is a requirement by Australian Institute of Family Studies, the custodian of the Longitudinal Study of Australian Children, to censor cell values with counts less than 10.

Among sexuality diverse female adolescents, 31% reported high levels of emotional symptoms, 56% reported probable depressive disorder, 42% reported past 12-month self-harm thoughts/behaviour and 27% reported past 12-month suicidal thoughts/behaviour. Compared with their heterosexual female peers, sexuality diverse females had increased odds of reporting high levels of emotional symptoms, probable depressive disorder, past 12-month self-harm thoughts/behaviour and past 12-month suicidal thoughts/behaviour (ORs ranged 2.2–2.6).

Among sexuality diverse male participants, 13% reported high levels of emotional symptoms, 30% reported probable depressive disorder, 14% reported past 12-month self-harm thoughts/behaviours and 13% reported past 12-month suicidal thoughts/behaviour. Sexuality diverse males had increased odds of high levels of emotional symptoms, probable depressive disorder and past 12-month suicidal thoughts/behaviour (ORs ranged 1.7–4.2).

### Substance use prevalence and disparities

Substance use varied between 66% and 97% among sexuality diverse participants; 73% and 97% among sexuality diverse female participants; and 41% and 93% among sexuality diverse male participants. Sexuality diverse females were over nine times as likely to report recent cannabis use (OR = 9.6, 95% confidence interval (CI): 1.1-85.4, *p* = *0.04*). There were no statistically significant differences in other substance use between sexuality diverse participants compared with non-sexuality diverse participants.

### Co-occurring mental ill-health and substance use disparities by sexuality

As shown in Table 3 below, sexuality diverse adolescents had significantly greater odds of co-occurring high levels of emotional symptoms and substance use, particularly alcohol use (OR = 3.7, 95% CI: 1.02–13.38, *p* *=* *0.04*); and cannabis use (OR = 6.2, 95% CI: 1.25–31.08, *p* *=* *0.02*). In addition, sexuality diverse adolescents had greater odds of co-occurring self-harm thoughts/behaviour and substance use, including alcohol use (OR = 7.6, 95% CI: 3.22–8.59, *p* *=* *0.05*) and cannabis use (OR = 9.3, 95% CI: 1.16–74.43, *p* *=* *0.03*).

**Table 3.** Disparities in co-occurring mental ill-health and substance use outcomes among sexuality diverse adolescents compared with non-sexuality diverse adolescents.

	Sexuality diverse *N* (%)	Non-sexuality diverse *N* (%)	OR, 95% CI	*p*
Outcome 1: Any mental ill-health and recent cigarette use				
No MH, No CU	*	36 (16.1)	1.0 (ref)	
MH only	*	35 (15.6)	3.4, 0.87–13.51	0.08
CU only	*	50 (22.3)	0.7, 0.14–3.77	0.70
MH and CU	22 (57.9)	103 (46.0)	2.6, 0.72–9.08	0.14
Outcome 2: Any mental ill-health and recent alcohol use				
No MH, No AU	*	11 (2.9)	1.0 (ref)	
MH only	*	13 (3.4)	1.7, 0.13–21.27	0.68
AU only	*	178 (46.4)	0.4, 0.05–3.83	0.45
MH and AU	50 (83.3)	182 (47.4)	3.0, 0.38–23.97	0.30
Outcome 3: Any mental ill-health and recent cannabis use				
No MH, No CBU	*	17 (10.9)	1.0 (ref)	
MH only	*	17 (10.9)	2.0, 0.17–24.13	0.59
CBU only	*	43 (27.6)	0.8, 0.07–9.27	0.85
MH and CBU	21 (80.8)	79 (50.6)	4.5, 0.57–35.81	0.15
Outcome 4: High levels of emotional symptoms and recent cigarette use				
No DE, No CU	*	60 (26.7)	1.0 (ref)	–
DE only	*	12 (5.3)	4.3 1.22–15.02	0.02
CU only	15 (39.5)	122 (54.2)	1.1 0.41–2.72	0.09
DE and CU	*	31 (13.8)	2.8 0.96–7.97	0.06
Outcome 5: High levels of emotional symptoms and recent alcohol use				
No DE, No AU	*	23 (6.0)	1.0 (ref)	
DE only	*	*	0	0.90
AU only	29 (48.3)	302 (78.6)	0.7, 0.21–2.60	0.6
DE and AU	28 (46.7)	58 (15.1)	3.7, 1.02–13.38	0.04
Outcome 6: High levels of emotional symptoms and recent cannabis use				
No DE, No CBU	*	30 (19.2)	1.0 (ref)	
DE only	*	*	3.7, 0.27–51.03	0.3
CBU only	13 (50.0)	98 (62.8)	2.0, 0.42–9.26	0.3
DE and CBU	*	24 (15.4)	6.2, 1.25–31.08	0.02
Outcome 7: Probable depressive disorder and recent cigarette use				
No DD, No CU	*	43 (19.2)	1.0 (ref)	
DD only	*	28 (12.5)	2.5, 0.73–8.28	0.14
CU only	*	68 (30.4)	0.5, 0.13–1.99	0.32
DD and CU	21 (55.3)	85 (37.9)	2.1, 0.75–6.02	0.15
Outcome 8: Probable depressive disorder and recent alcohol use				
No DD, No AU	*	13 (3.4)	1.0 (ref)	
DD only	*	11 (2.9)	0.6, 0.05–7.43	0.68
AU only	12 (20.0)	213 (55.5)	0.4, 0.07–1.81	0.21
DD and AU	45 (75.0)	23147 (38.3)	2.0, 0.43–9.15	0.37
Outcome 9: Probable depressive disorder and recent cannabis use				
No DD, No CBU	*	20 (12.8)	1.0 (ref)	
DD only	*	14 (9.0)	0.7, 0.06–8.66	0.79
CBU only	*	59 (37.8)	0.5, 0.08–3.27	0.47
DD and CBU	20 (76.9)	20 (76.9)	3.2, 0.68–14.78	0.14
Outcome 10: Self-harm thoughts/behaviour and recent cigarette use				
No SH, No CU	*	52 (23.2)	1.0 (ref)	
SH only	*	19 (8.5)	4.4, 1.27–15.05	0.01
CU only	*	94 (42.0)	0.9, 0.28–2.84	0.83
SH and CU	16 (43.2)	59 (26.3)	2.8, 0.97–8.23	0.057
Outcome 11: Self-harm thoughts/behaviour and recent alcohol use				
No SH, No AU	*	19 (4.9)	1.0 (ref)	
SH only	*	*	7.6, 0.57–101.69	0.12
AU only	16 (27.1)	260 (67.7)	1.2, 0.15–9.28	0.88
SH and AU	40 (67.8)	100 (26.0)	7.6 3.22–8.59	0.05
Outcome 12: Self-harm thoughts/behaviour and recent cannabis use				
No SH, No CBU	*	25 (16.0)	1.0 (ref)	
SH only	*	*	5.6, 0.45–68.94	0.18
CBU only	*	79 (50.6)	1.9, 0.22–16.53	0.56
SH and CBU	16 (64.0)	16 (64.0)	9.3, 1.16–74.43	0.03
Outcome 13: Suicide thoughts/behaviour and recent cigarette use				
No SB, No CU	*	58 (25.9)	1.0 (ref)	
SB only	*	13 (5.8)	2.8, 0.78–9.92	0.11
CU only	10 (27.0)	98 (43.8)	0.7, 0.28–1.98	0.54
SB and CU	14 (37.8)	55 (24.6)	1.8, 0.66–1.37	0.20
Outcome 14: Suicide thoughts/behaviour and recent alcohol use				
No SB, No AU	*	19 (4.9)	1.0 (ref)	
SB only	*	*	0.0	0.9
AU only	29 (49.2)	279 (72.7)	0.7, 0.18–2.36	0.52
SB and AU	27 (45.8)	81 (21.1)	2.1, 0.58–7.70	0.25
Outcome 15: Suicide thoughts/behaviour and recent cannabis use				
No SB, No CBU	*	26 (16.7)	1.0 (ref)	
SB only	*	*	1.6, 0.13–20.36	0.7
CBU only	*	82 (52.6)	1.4, 0.29–7.03	0.66
SB and CBU	13 (52.0)	40 (25.6)	4.2, 0.88–20.28	0.07

OR: odds ratio; CI: confidence interval; AU: alcohol use, CU: cigarette use, CBU: cannabis use; DE: high levels of emotional symptoms; DD: probable depressive disorder; MH: any mental ill-health outcome (i.e. probable depressive disorder, high levels of emotional symptoms, self-harm thoughts/behaviour, suicide thoughts/behaviour); SH: self-harm thoughts/behaviour; SB: suicide thoughts/behaviour.

* Denote counts under 10, which are required by Australian Institute of Family Studies policy to be censored in data reporting and presentation.

### School climate and sexuality diverse adolescent mental ill-health and substance use

Among sexuality diverse adolescents, greater school climate scores were significantly associated with lower odds of reporting probable anxiety/depressive disorder, probable depressive disorder, self-harm thoughts/behaviours and suicidal thoughts/behaviour (OR = 0.9, 95% CI: 0.86–0.92, *p* *<* *0.0001*; OR = 0.9, 95% CI: 0.85–0.91, *p* *<* *0.0001*; OR = 0.9, 95% CI: 0.87–0.93, *p* *<* *0.0001*; OR = 0.9, 95% CI: 0.88–0.94, *p* *<* *0.001*; respectively). For each one-point increase in school climate scores as measured by the PSSM, there was a 10% reduction of sexuality diverse adolescents reporting a probable anxiety/depressive disorder, probable depressive disorder, self-harm thoughts/behaviours and suicidal thoughts/behaviour. These findings are shown in Table 4 below.

**Table 4.** Associations between school climate with mental health and substance use among sexuality diverse adolescents.

	OR 95% CI	*p*
High levels of emotional symptoms	0.9 0.86–0.92	< 0.0001
Probable depressive disorder	0.9 0.85–0.91	< 0.0001
Past 12-month self-harm thoughts and behaviours	0.9 0.87–0.93	< 0.0001
Past 12-month suicidal thoughts and behaviours	0.9 0.88–0.94	< 0.0001
Past 12-month cigarette use	1.2 1–1.3	0.01
Past 12-month alcohol use	1.0 1.0–1.1	0.20
Past 12-month cannabis use	1.0 0.8–1.3	0.75
Any mental ill-health and past 12-month cigarette use	1.1 0.9–1.2	0.45
Any mental ill-health and past 12-month alcohol use	1.0 0.9–1.0	0.12
Any mental ill-health and past 12-month cannabis use	1.0 0.8–1.3	0.74

OR: odds ratio; CI: confidence interval.

### Teacher self-efficacy and sexuality diverse adolescent mental ill-health and substance use

Among the teachers of sexuality diverse participants within this sample, lower perceived teacher self-efficacy was significantly associated with greater odds of sexuality diverse student-reported recent suicidal thoughts/behaviours (OR = 1.8, 95% CI: 1.06–3.23, *p* *=* *0.03*). Improved teacher self-efficacy was associated with past 12-month cannabis use (OR = 0.1, 95% CI: 0.0–0.9, *p* = *0.04*) and any mental ill-health and past 12-month cannabis use (OR = 0.1, 95% CI: 0.0–0.9, *p* = *0.04*). These findings are reported in full in Table 5 below.

**Table 5.** Associations between teacher self-efficacy and mental health and substance use outcomes among sexuality diverse adolescents.

	OR 95% CI	*p*
High levels of emotional symptoms	1.4 0.82–2.38	0.22
Probable depressive disorder	1.4 0.84–2.21	0.2
Past 12-month self-harm thoughts and behaviours	1.7 0.98–3.00	0.06
Past 12-month suicidal thoughts and behaviours	1.8 1.06–3.23	0.03
Past 12-month cigarette use	1.4 0.1–13.3	0.77
Past 12-month alcohol use	1.0 0.4–2.4	0.94
Past 12-month cannabis use	0.1 0.0–0.9	0.04
Any mental ill-health and past 12-month cigarette use	1.4 0.1–13.3	0.77
Any mental ill-health and past 12-month alcohol use	1.0 0.5–2.3	0.94
Any mental ill-health and past 12month cannabis use	0.1 0.0–0.9	0.04

OR: odds ratio; CI: confidence interval.

## Discussion

This study is the first to use population-level, nationally representative data to estimate the prevalence and disparities in mental ill-health, substance use and their co-occurrence, among sexuality diverse adolescents in Australia. This study is also the first to show preliminary evidence regarding the preventive utility of school climate and teacher self-efficacy for preventing and reducing mental ill-health and co-occurring substance use.

Our analyses identified alarming disparities in mental ill-health experiences by sexuality diverse adolescents, particularly sexuality diverse female adolescents. This provides further evidence that mental ill-health disparities are emergent during early adolescence, an important distinction from past research, which considers mental ill-health disparities among sexuality diverse young people broadly through adolescence and young adulthood (Gilbey et al., 2020; Hill et al., 2021b). These findings extend existing literature, which largely focuses on sex-differences in mental health among the general adolescent population (Campbell et al., 2021), or sexuality diverse adults (MacCarthy et al., 2021).

Sexuality diverse females were significantly more likely to use cannabis compared to heterosexual females, providing new evidence that previously identified disparities in cannabis use documented among sexuality diverse adult populations (Schofield et al., 2023) may, in fact, emerge before or during early adolescence. Urgent research coupled with education about the potential harms of cannabis use during adolescence is required to better understand and prevent cannabis use among sexuality diverse adolescents, given cannabis use is a significant risk factor for cannabis use disorder and associated psychiatric comorbidity (Teesson et al., 2012). Better understanding the impact of effective substance use prevention programmes that go beyond education provision is one possible pathway towards addressing this gap (Andrews et al., 2023; Thornton et al., 2018). It is hypothesised that cannabis use may be a coping mechanism for minority stress, that is, how heterosexist society privileges heterosexual people to stigma, discrimination and subsequent mental ill-health detriment of sexuality diverse people (Buttazzoni et al., 2021; Meyer, 2003). Additional public health efforts are required to address the complex pathways between minority stress and cannabis use among sexuality diverse adolescents with the view to preventing and reducing cannabis use as a potentially harmful coping mechanism (Buttazzoni et al., 2021; Schofield et al., 2023). Furthermore, due to relatively low response rates to substance use items, albeit largely system missing, future research on substance use disparities among sexuality diverse adolescents would benefit from leveraging large sample sizes to improve confidence and precision in estimates.

Our study illustrates the disproportionate burden of co-occurring mental ill-health and substance use affecting sexuality diverse adolescents, particularly the potential use of multiple different substances in co-occurrence with high levels of emotional symptoms and self-harm thoughts/behaviour. These findings represent a significant and novel contribution to the literature given most previous research is largely limited to sexuality diverse adults (Felner et al., 2021; Han et al., 2020) or individual mental ill-health and/or substance use as siloed public health issues (Scheer et al., 2022; Watson et al., 2020). While part of our findings may be explained as sexuality diverse adolescents using substances to cope with sexuality diversity-related minority stress and associated and/or independent symptoms of depression and anxiety (Felner et al., 2020; Johannessen et al., 2017), co-occurring substance use and self-harm thoughts/behaviours pose a slightly more complicated public health issue. Self-harm is commonly documented in the literature as being a coping mechanism in and of itself, for mental ill-health and life adversity (Guerreiro et al., 2015; Klineberg et al., 2013). Moreover, self-harm thoughts/behaviours during adolescence are strong predictors of substance use and misuse through adolescence and adulthood (Moran et al., 2015). Further research and tailored community-engaged public health support is required to better understand experiences of sexuality diverse adolescents coping with mental ill-health, particularly those with self-harm thoughts/behaviours and/or substance use. Without further preventive research and intervention, co-occurring mental ill-health and substance use among sexuality diverse adolescents, particularly that involving self-harm thoughts/behaviours, renders this population at significantly higher risk of chronic, debilitating substance use disorders and significant psychiatric morbidity across the lifespan.

Importantly, positive school climate appears to buffer and reduce mental ill-health among sexuality diverse adolescents within this sample, in the early to middle stages of their schooling experiences. This highlights a highly feasible way to improve efforts to support sexuality diverse young people within the school environment. Past research often defines school climate with respect to sexuality diverse young people as the presence or absence of sexuality diverse-specific programmes or policies, such as GSAs or anti-bullying policies, yet these measures fail to capture the totality of school as a physical and cultural institution that deeply influences how students perceived themselves and their relationships with peers and staff (Colvin et al., 2019; Day et al., 2020; Leung et al., 2022; Parodi et al., 2022). Hence, our findings provide robust evidence for the necessity and public health preventive value of whole-of-school approaches that are affirming, inclusive and supportive of sexuality diverse young people, especially those in early adolescence, to combat already emergent and pronounced mental ill-health disparities. These school-wide approaches are defined as predictable, reliable routines conducive of affirming and including sexuality diverse adolescents, including the provision of information and support relating to sexuality diversity to all students, school-wide teacher professional development and inclusive curricula (Russell et al., 2021).

Our study also provides preliminary evidence that teacher self-efficacy indeed plays a significant role in sexuality diverse mental ill-health, specifically during early adolescence. Sexuality diverse adolescents are at significantly increased risk of bullying and harassment compared to their heterosexual peers (Ullman, 2021). Further research is required to understand whether teacher self-efficacy is associated with positive adult intervention in these adverse experiences. Notwithstanding this, these findings point to an important evidence-based policy recommendation that teachers and other school practitioners should undergo formal training and professional development tailored specifically to affirming, supporting and including sexuality diverse adolescents. Current Australia-specific findings suggest gender and sexuality diversity is the subject teachers and other school staff feel least comfortable teaching and discussing with students (Ezer et al., 2021). Within the wider school climate, teachers specifically play an important role in preventing and reducing mental ill-health among sexuality diverse adolescents and young people. Additional research should also identify whether teacher self-efficacy is associated with mental ill-health among trans, non-binary and gender-diverse young people (those adolescents with a gender identity different to the gender presumed for them at birth, henceforth respectfully referred to using the umbrella term, ‘trans’) given their relatively higher burden of mental ill-health. It is also important to note that the teacher self-efficacy and school climate measures were not specific to sexuality diversity nor were the religion of participants or their respective schools examined. Hence all schools including government, private and religious schools should be looking to promote teacher self-efficacy in managing behavioural and learning problems and improving school climate to ensure all young people feel they can be themselves at school.

Whereas our study benefitted from significant strengths such as a large, nationally representative, cohort of sexuality diverse adolescents, a comprehensive assessment of mental ill-health symptomatology and recent substance use, and the use of both student- and teacher-report measures, this study is not without limitations. Missing data on substance use and teacher self-efficacy, albeit largely system missing in nature, may have biased the external validity of the sample, particularly if teacher non-respondents were less likely to possess self-efficacy about their teaching abilities. Future research drawing on both student- and teacher-reports of mental health and school climate should capture comprehensive demographic and psycho-social influences for both the adolescent participant but also their teacher, to ensure any missing data can be subject to rigorous sensitivity analyses. While the school climate measure utilised in this study is highly reliable and valid, its focus is on the adolescent’s perceived sense of belonging within school, which does not incorporate other facets of school climate, including safety/discipline, equality of opportunity and environment (Gonzálvez et al., 2023). Moreover, the PSSM scale does not include specific items relating to the level of sexuality diversity affirmative and inclusive measures in schools, a likely important factor imperative for contemporary studies of school climate and especially sexuality diverse adolescent’s perceptions of school climate. Future research should draw on sexuality diverse specific measures of school climate, such as the recently validated Gender Climate scale (Ullman et al., 2023), to strategically determine which aspects of school climate can best be modified to promote mental health among sexuality diverse adolescents. Due to limited sample size, analyses of school climate and teacher self-efficacy associations with co-occurring mental ill-health and substance use outcomes should be interpreted with caution. Furthermore, this limitation may also explain the unexpected finding of an association between past 12-month cannabis use and improved teacher self-efficacy, and any mental ill-health and past 12-month cannabis use and improved teacher self-efficacy. Further research harnessing large sample sizes of sexuality-diverse adolescents and their heterosexual peers are required to establish the true effect estimate of the relationship between school climate, teacher self-efficacy and mental ill-health and substance use outcomes. The four bespoke items assessing teacher self-efficacy were also a limited qualification of teachers’ true sense of efficacy despite generating an acceptable level alpha co-efficient. Moreover, it was unclear whether some participants shared teachers. While analyses adjusted for geographical postcode-level clustering, there may have been residual confounding if there was a potential lack of independence of observations. Future relevant research should incorporate teachers’ personal values and motivations for teaching (Barni et al., 2019), particularly in relation to sexuality diversity and inclusive education, to understand how these can be modified for the benefit of adolescent mental health. Another limitation of the study relates to how this wave of the LSAC study did not include gender indicators, preventing any analyses on trans, non-binary and gender diverse adolescents. Trans adolescents experience vastly greater mental ill-health disparities compared with their cisgender peers. Rates of mental ill-health among trans adolescents are typically observed as higher than those among sexuality diverse adolescents. Future research leveraging nationally representative, population-level sampling methodologies should ensure that adequate gender indicators are included to enable high-quality public health research to promote trans mental health.

In summation, mental ill-health and substance use, and especially their co-occurrence, are highly prevalent among sexuality diverse adolescents in Australia and pose significant and inequitable risks to health and well-being through adolescence and across the lifespan. Urgent research and community-sensitive preventive education is required to address this critical public health issue. Schools represent a potential site for focusing these prevention efforts. Educating and training teachers is a promising pathway towards optimising school-based prevention.
